# Device and surgical procedure-related infections in Canadian acute care hospitals from 2011 to 2020

**DOI:** 10.14745/ccdr.v48i78a04

**Published:** 2022-07-07

**Authors:** Canadian Nosocomial Infection Surveillance Program

**Affiliations:** 1 Centre for Communicable Diseases and Infection Control, Public Health Agency of Canada, Ottawa, ON

**Keywords:** hospital-associated infection, acute care, surveillance, antimicrobial resistance, device-associated infection, surgical procedure-related infection, surgical site infections, Canada

## Abstract

**Background:**

Healthcare-associated infections (HAIs) continue to place a burden on patient health and safety as well as on the healthcare system. In Canada, national surveillance of HAIs at sentinel acute care hospitals is conducted by the Canadian Nosocomial Infection Surveillance Program. This article describes ten years of device and surgical procedure-related HAI epidemiology in Canada from 2011 to 2020.

**Methods:**

Data were collected from over 40 Canadian sentinel acute care hospitals between January 1, 2011, and December 31, 2020, for central line-associated bloodstream infections (CLABSIs), hip and knee surgical site infections (SSIs), cerebrospinal fluid shunt SSIs and paediatric cardiac SSIs. Case counts, rates, patient and hospital characteristics, pathogen distributions, and antimicrobial resistance are presented.

**Results:**

Between 2011 and 2020, 4,751 device and surgical procedure-related infections were reported, with CLABSIs in intensive care units (ICUs) representing 67% (n=3,185) of all reported infections. Over the surveillance period, significant rate increases were observed in adult mixed ICU CLABSIs (0.8 to 1.6 per 1,000 line days, *p*=0.004) while decreases were observed in neonatal ICU CLABSIs (4.0 to 1.6 per 1,000 line days, *p*=0.002) and SSIs following knee arthroplasty (0.69 to 0.29 infections per 100 surgeries, *p*=0.002). No trends were observed in the other reported HAIs.

Of the 5,071 pathogens identified, the majority were gram-positive (68%), followed by gram-negative (23%) and fungi (9%). Coagulase-negative staphylococci (27%) and *Staphylococcus aureus* (16%) were the most frequently isolated pathogens.

**Conclusion:**

This report describes epidemiological and microbiological trends among select device and surgical procedure-related HAIs, essential for benchmarking infection rates nationally and internationally, to identify any changes in infection rates or antimicrobial resistance patterns and to help inform hospital infection prevention and control and antimicrobial stewardship policies and programs.

## Introduction

Healthcare-associated infections (HAIs) threaten patient safety and quality of care, contributing to prolonged hospital stays, increased antimicrobial resistance, costs to the health system and unnecessary deaths ((1)). Healthcare-associated infections may arise through the use of invasive devices, surgical procedures and inappropriate antibiotic use ((2)). A 2017 point prevalence study at Canadian sentinel acute care hospitals found that device and surgical procedure-related infections accounted for 35.6% of all reported HAIs ((3)). Among these device and surgical procedure-related infections, 19.4% of surgical site infections (SSIs) were associated with a prosthetic implant while 21.2% were associated with central line-associated bloodstream infections (CLABSIs) ((3)). The risk of device and surgical procedure-related HAIs varies among patient populations and within hospital types, with patients admitted to the intensive care unit (ICU) being at higher risk of developing a HAI ((4)). During the coronavirus disease 2019 (COVID-19) pandemic declared by the World Health Organization on March 11, 2020 ((5)), rates of HAIs and antimicrobial resistance (AMR) may have been impacted by necessary changes to hospital infection prevention and control practices and antimicrobial stewardship ((6)).

Antimicrobial resistance is known to impact length of stay and healthcare costs ((7)). It is expected that by 2050 an estimated 10 million annual deaths will be attributable to AMR ((8)); thus, antimicrobial susceptibility information is key to ensuring appropriate treatment and use of antimicrobials to help reduce AMR ((9)).

Understanding the trends in device and surgical procedure-related HAIs is essential to provide benchmark rates over time which helps to inform effective antimicrobial stewardship and infection prevention and control measures. This report provides an epidemiological overview of select device and surgical procedure-related HAIs from 2011 to 2020 in over 40 Canadian Nosocomial Infection Surveillance Program (CNISP) hospitals.

## Methods

### Design

Since its establishment in 1994, CNISP has conducted national HAI surveillance at sentinel acute care hospitals across Canada, in collaboration with the Public Health Agency of Canada and the Association of Medical Microbiology and Infectious Disease Canada. Data are presented for the following device and surgical procedure-related HAIs: central line-associated bloodstream infections (CLABSIs); hip and knee arthroplasty SSIs; cerebrospinal fluid (CSF) shunt SSIs; and paediatric cardiac SSIs.

### Case definitions

Device and surgical procedure-related HAIs were defined according to standardized protocols and expert-reviewed case definitions (see **Appendix**). Only complex infections, defined as deep incisional and organ/space, were included in hip and knee SSI surveillance, while only CLABSIs identified in ICU settings. Adult mixed ICU, adult cardiovascular surgery intensive care unit (CVICU), paediatric intensive care unit (PICU) and neonatal intensive care unit (NICU) were included in CLABSI surveillance.

### Data source

Epidemiological data on device and surgical procedure-related infections occurring between January 1, 2011 and December 31, 2020 were submitted by participating hospitals. Data submission and case identification were supported by training sessions and periodic evaluations of data quality.

### Statistical analysis

To calculate hip and knee SSI, CSF shunt SSI and paediatric cardiac SSI rates, the number of cases were divided by the number of surgical procedures performed (multiplied by 100). To calculate CLABSI rates, the number of cases were divided by line day denominators (multiplied by 1,000). To calculate proportions of pathogens, the number of pathogens were divided by the total number of identified pathogens. Denominators may vary, as missing and incomplete data were excluded from analyses. Interquartile ranges (IQR) were calculated. Trends over time were tested using the Mann-Kendall test. Significance testing was two-tailed and differences were considered significant at a *p*-value of ≤0.05. Analyses were conducted using R version 4.1.2 and SAS 9.4.

## Results

Over 40 hospitals contributed device and surgical procedure-related infection data to CNISP between 2011 and 2020, most of which were medium (201−499 beds) adult hospitals (**Table 1**). Overall, 4,751 device and surgical procedure-related infections were reported. Among all reported HAIs, CLABSIs were the most common representing 67% (n=3,185) of all device and surgical procedure-related HAIs. Among all SSIs reported (N=1,566), hip and knee infections represented 70% (n=1,093).

**Table 1:** Characteristics of acute care hospitals participating in device and surgical procedure-related healthcare-associated infection surveillance, 2011–2020

Characteristic of hospitals	CLABSI- adult mixed ICU	CLABSI- adult CVICU	CLABSI-PICU	CLABSI-NICU	CSF shunt SSI	Paediatric cardiac SSI	Hip and knee SSI
Number of HAIs reported	1,544	200	396	1,045	239	234	1,093
Total number of participating hospitals	31–40	6–9	9–12	15–19	11–15	4–5	12–28
Hospital type							
Adult	21–29	5–8	N/A	3–4a	3–4	N/A	8–16
Mixed	9–13	1–2	4	4–6	2–3	N/A	4–13
Paediatric	N/A	N/A	5–8	6–9	6–8	4–5	N/A
Hospital size							
Small (1–200 beds)	2–5	0–1	4–8	5–10	5–6	4	0–2
Medium (201–499 beds)	19–27	3–4	3–5	5–8	4–6	0–1	7–18
Large (500+ beds)	9–12	3–4	0–1	1–4	2–3	N/A	5–8

Abbreviations: CLABSI, central line-associated bloodstream infection; CSF shunt SSI, cerebrospinal fluid shunt surgical site infection; CVICU, cardiovascular surgery intensive care unit; HAIs, healthcare-associated infections; ICU, intensive care unit; N/A, not applicable; NICU, neonatal intensive care unit; PICU, paediatric intensive care unit; SSI, surgical site infection

^a^ Four hospitals classified as “adult” also had a NICU

A total of 5,071 pathogens were identified from device and surgical procedure-related HAI cases between 2011 and 2020. Coagulase-negative staphylococci (CoNS) and *Staphylococcus aureus* were the most frequently reported pathogens (**Table 2**). Of the identified pathogens, 67.7% were gram-positive, 23.0% were gram-negative and 9.3% were fungal.

**Table 2:** Distribution and rank of the five most frequently reported gram-negative, gram-positive and fungal pathogens, 2011–2020^a^

Pathogen category	Rank	Pathogen	CLABSI N=3,185		Hip and knee N=1,093			CSF shunt N=239				Paediatric cardiac N=234		Total pathogens	
			n	%	n	%		n		%		n	%	n	%
Gram-positive	1	Coagulase-negative staphylococci^b^	991	28.6	218	18.2		99		40.1		36	22.2	1,344	26.5
	2	*Staphylococcus aureus* ^c^	268	7.7	381	31.8		59		23.9		77	47.5	785	15.5
	3	*Enterococcus* spp.	523	15.1	84	7.0		14		5.7		1	0.6	622	12.3
	4	*Streptococcus* spp.	63	1.8	106	8.9		6		2.4		11	6.8	186	3.7
	5	Methicillin-resistant *S. aureus*	67	1.9	79	6.6		9		3.6		9	5.6	164	3.2
	Other gram-positive^d^		206	5.9	104	8.7		21		8.5		1	0.6	332	6.5
	Total gram-positive		2,118	61.1	972	81.2		208		84.2		135	83.3	3,659	67.7
Gram-negative	1	*Klebsiella* spp.	235	6.8	22	1.8		3		1.2		0	0.0	260	5.1
	2	*Escherichia coli*	183	5.3	32	2.7		10		4.0		2	1.2	227	4.5
	3	*Enterobacter* spp.	154	4.4	43	3.6		4		1.6		3	1.9	204	4.0
	4	*Pseudomonas* spp.	93	2.7	51	4.3		10		4.0		4	2.5	158	3.1
	5	*Serratia* spp.	83	2.4	15	1.3		2		0.8		3	1.9	103	2.0
	Other gram-negative^e^		150	4.3	57	4.8		5		2.0		3	1.9	215	4.2
	Total gram-negative		898	25.9	220	18.4		34		13.8		15	9.3	1,167	23.0
Fungi	1	*Candida albicans*	212	6.1	0	0.0		1		0.4		1	0.6	214	4.2
	2	Other *Candida* spp.^f^	221	6.4	4	0.3		2		0.8		8	4.9	235	4.6
	Other fungi^g^		16	0.5	1	0.1		2		0.8		3	1.9	22	0.4
	Total fungal		449	13.0	5	0.4		5		2.0		12	7.4	471	9.3
Total			3,465	3,465	1,197		1,197		247		247	162	162	5,071^h^	5,071^h^

Abbreviation: CLABSI, central line-associated bloodstream infections

^a^ Frequency distribution percentage rounded to the nearest tenth decimal

^b^ Coagulase-negative staphylococci included *S. lugdunensis*, *S. haemolyticus*, *S. epidermidis*, *S. capitis*, *S. hominis* and *S. warneri*

^c^ *Staphylococcus aureus* includes methicillin-susceptible *S. aureus* and unspecified *S. aureus*

^d^ Other gram-positive pathogens included anaerobic gram-positive cocci, *Finegoldia magna*, *Clostridioides* spp., *Lactobacillus* spp. and others

^e^ Other gram-negative pathogens included *Stenotrophomonas* spp., *Morganella morganii*, *Proteus mirabilis*, *Prevotella* spp., *Bacteroides fragilis* and others

^f^ Other *Candida* spp. included *C. dubliniensis*, *C. glabrata*, *C. krusei*, *C. lusitaniae*, *C. parapsilosis* and *C. tropicalis*

^g^ Other fungi included *Aspergillus* spp., *Trichophyton tonsurans* and yeast

^h^ Up to three pathogens per device and surgical procedure-related infection were included in the analysis and exceeded the number of total reported infections overall

### Central line-associated bloodstream infections

A total of 3,185 CLABSIs were reported between 2011 and 2020, with the majority occurring in adult mixed ICUs (n=1,544, 48.5%) and NICUs (n=1,045, 32.8%). Overall, NICUs had the highest rates of CLABSIs between 2011 and 2020 (2.3 infections per 1,000 line days), followed by PICUs (1.6 per 1,000 line days), adult mixed ICUs (1.1 per 1,000 line days) and adult CVICUs (0.6 per 1,000 line days) (**Table A1**).

**Figure 1:**
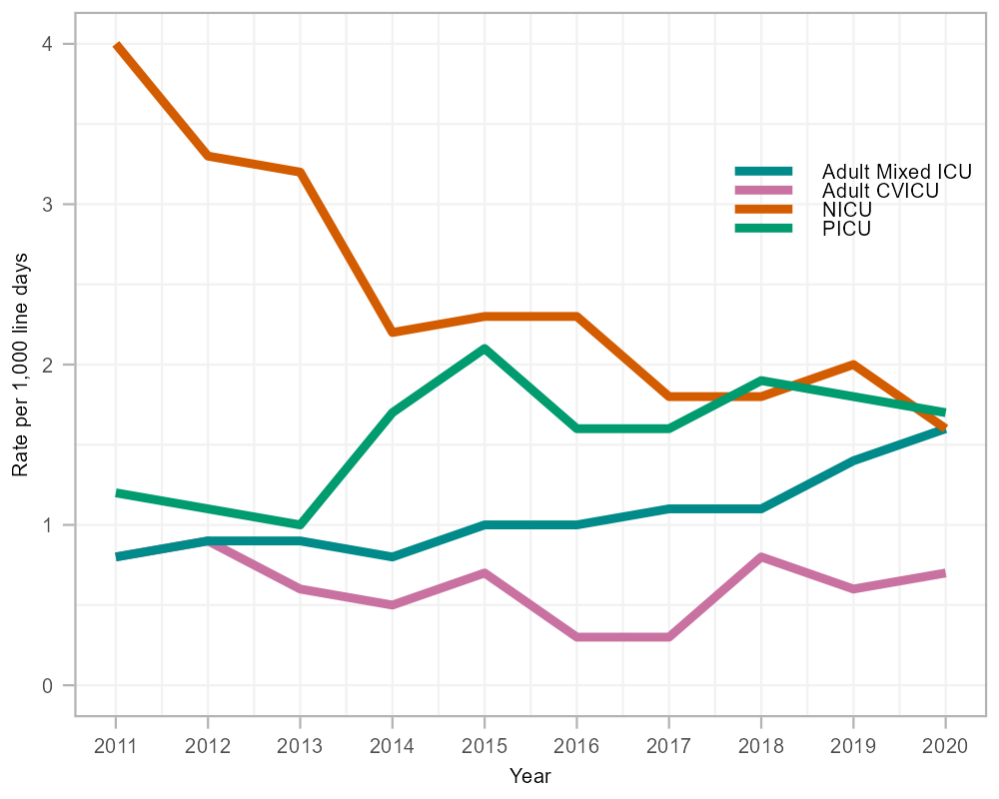
Rate of central line-associated bloodstream infection per 1,000 line days by intensive care unit type, 2011–2020 Abbreviations: CLABSI, central line-associated bloodstream infection; CVICU, cardiovascular intensive care unit; ICU, intensive care unit; NICU, neonatal intensive care unit; PICU, paediatric intensive care unit

While CLABSI rates fluctuated in PICUs and adult CVICUs, adult mixed ICU CLABSI rates doubled between 2011 and 2020 (0.8 to 1.6 infections per 1,000 line days, *p*=0.004) (**Figure 1**), driven by the Central region (Ontario and Québec) since 2015 and the Western region (British Columbia, Alberta, Saskatchewan and Manitoba) since 2017 (data not shown). Concomitantly, a 60% rate decrease was observed in NICU CLABSIs (4.0 to 1.6 infections per 1,000 line days, *p*=0.002). Compared to 2019, CLABSI rates in 2020, during the COVID-19 pandemic, followed similar trends to those observed since 2011; adult mixed ICU CLABSIs continued to increase (14%, 1.4 to 1.6 infections per 1,000 line days) and NICU CLABSIs decreased (20%, 2.0 to 1.6 infections per 1,000 line days), while adult CVICU and PICU CLABSIs remained stable.

Among CLABSIs identified in adult mixed ICUs, the median age was 61 years (IQR=48–71 years), with males representing 61.6% of cases. All-cause mortality within 30 days following the first positive culture, for adult mixed ICU CLABSI patients was 32.2% (n=491/1,524). Among CLABSIs identified in adult CVICUs, the median age was 66 years (IQR=56–73 years), with males representing 69.0% of cases. Within 30 days following the first positive culture, all-cause mortality for adult CVICU CLABSI patients was 31.5% (n=62/197). Among CLABSIs identified in PICUs, the median age was six months (IQR=2−28 months), with males representing 55.6% of cases. Within 30 days following the first positive culture, all-cause mortality for PICU CLABSI patients was 9.6% (n=38/396). Among CLABSIs identified in NICUs, the median age at first positive culture was 17 days (IQR=9−47 days). Males represented 58.6% of NICU cases and all-cause mortality within 30 days of positive culture was 9.2% (n=96/1,043).

The most commonly identified pathogens among CLABSIs overall were CoNS and *Enterococcus* spp. (28.5% and 15.0%, respectively), which aligned with the most commonly identified pathogens among PICUs and adult CVICUs. Among adult mixed ICUs and NICU CLABSIs, CoNS and *S. aureus* were the most commonly identified pathogens.

### Hip and knee surgical site infections

**Figure 2:**
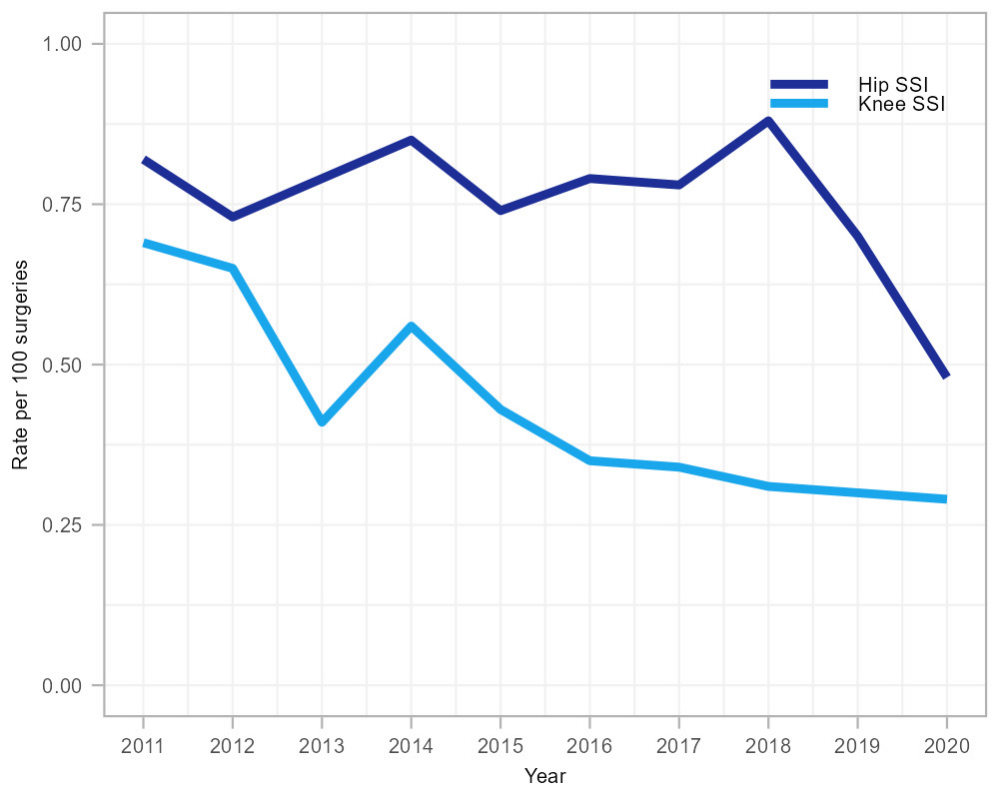
Rate of hip and knee surgical site infections per 100 surgeries, 2011–2020

A total of 1,093 complex hip and knee SSIs were reported between 2011 and 2020, the majority (n=672, 61.5%) among hip arthroplasties. Among hip and knee SSIs, 51.7% (n=565) were organ/space infections and 48.3% (n=528) were deep incisional infections (**Table 3**). From 2011 to 2020, knee SSI rates decreased significantly (58.0%, 0.69 to 0.29 infections per 100 surgeries, *p*=0.002) while hip SSI rates fluctuated between 0.48 and 0.88 infections per 100 surgeries (*p*=0.33). Hip SSI rates decreased 31% in 2020 compared to rates observed in 2019 (0.70 to 0.48 infections per 100 surgeries) while knee SSI rates remained stable (**Figure 2** and **Table A2**).

**Table 3:** Frequency of hip and knee surgical site infections by year and infection type, 2011–2020

Year	Deep incisional SSI		Organ/space SSI		All cases
	n	%	n	%	n
Hip arthroplasty					
2011	18	43.9	23	56.1	41
2012	32	66.7	16	33.3	48
2013	36	57.1	27	42.9	63
2014	36	50.7	35	49.3	71
2015	34	52.3	31	47.7	65
2016	28	41.2	40	58.8	68
2017	34	42.0	47	58.0	81
2018	34	34.7	64	65.3	98
2019	46	51.1	44	48.9	90
2020	22	46.8	25	53.2	47
Overall	320	47.6	352	52.4	672
Knee arthroplasty					
2011	20	51.3	19	48.7	39
2012	26	52.0	24	48.0	50
2013	21	55.3	17	44.7	38
2014	26	48.1	28	51.9	54
2015	21	47.7	23	52.3	44
2016	15	41.7	21	58.3	36
2017	18	43.9	23	56.1	41
2018	22	55.0	18	45.0	40
2019	25	53.2	22	46.8	47
2020	14	43.8	18	56.3	32
Overall	208	49.4	213	50.6	421

Abbreviation: SSI, surgical site infection

The median patient age was 68 years (IQR=59–77 years) for hip SSIs and 66 years (IQR=60–74 years) for knee SSIs. The median time from procedure to hip and knee infections was 21 days (IQR=14–32 days) and 23 days (IQR=14–35 days), respectively. For complex SSIs following hip and knee arthroplasties, the median length of stay was 3 days (IQR=2–6 days). Data collected between 2018 and 2020 indicate that 90.6% of patients with an SSI following hip or knee arthroplasty were readmitted (hip: n=211/233, 90.6%; knee: n=108/119, 90.8%) and 67.2% (n=231/344) required revision surgery. Within 30 days after first positive culture, four all-cause deaths (1.8%, n=4/225) were reported among patients with a complex SSI following a hip arthroplasty while zero were reported following a knee arthroplasty SSI. Among hip and knee SSI cases, *S. aureus* and CoNS were the most commonly identified pathogens at 32% and 18%, respectively, and did not differ by deep or organ/space infection type (data not shown).

### Cerebrospinal fluid shunt surgical site infections

**Figure 3:**
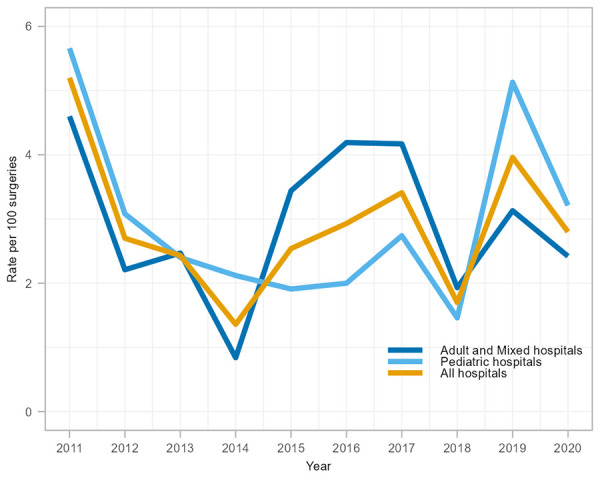
Cerebrospinal fluid shunt surgical site infection rates per 100 surgeries by hospital type^a^, 2011–2020 ^a^ All hospitals include adult, mixed, and paediatric hospitals participating in cerebrospinal fluid shunt surgical site infection surveillance

Between 2011 and 2020, 239 CSF shunt SSIs were reported, with an overall rate of 2.9 infections per 100 surgeries (range: 1.4 to 5.2 infections per 100 surgeries, **Table A3**). Paediatric and adult/mixed hospitals had similar infection rates at 3.0 and 2.8 infections per 100 surgeries, respectively. In 2020, CSF shunt SSI rates decreased compared to 2019 (28%, 4.0 to 2.9 infections per 100 surgeries); however, this decrease was in keeping with the fluctuating rate trend since 2011 (**Figure 3**).

More than half of CSF shunt SSIs (55.6%, n=130/234) were identified from new surgeries while 44.4% (n=104/234) were identified from revision surgeries. The median age was 47 years (IQR=34–60 years) for adult patients and 0.9 years (IQR=0.2–6.6 years) for paediatric patients. Females represented 52.3% (n=123/235) of cases and median time from surgery to infection was 21 days (IQR=12–43 days). The most commonly identified pathogens from CSF shunt SSIs were CoNS and *S. aureus* (40% and 24% of identified pathogens, respectively). Outcome data are not collected for CSF shunt SSI surveillance.

### Paediatric cardiac surgical site infections

**Figure 4:**
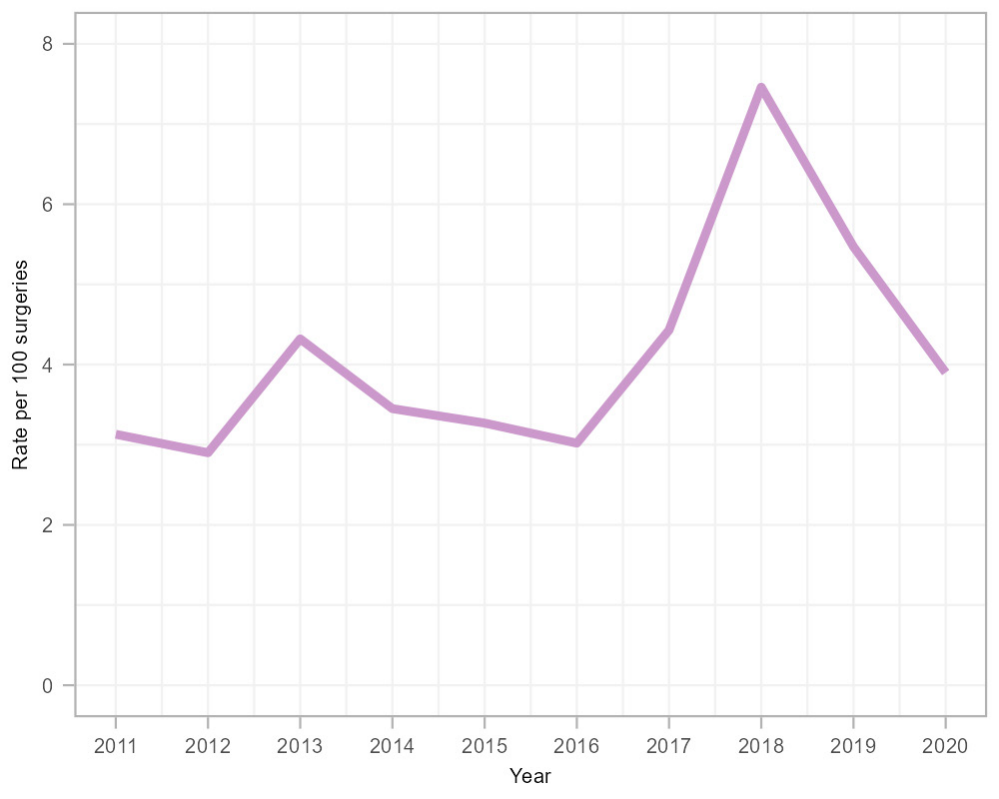
Paediatric cardiac surgical site infection rates per 100 surgeries, 2011–2020

A total of 234 paediatric cardiac SSIs were reported between 2011 and 2020 (**Table 4**), most of which were superficial infections (63.1%). Organ/space infections accounted for 29.2% of these SSIs. Overall, the average paediatric cardiac SSI rate was 4.1 infections per 100 surgeries (**Table A4**). While rates remained generally consistent over the surveillance period (*p*=0.089), there was a significant increase in 2018 (7.5 infections per 100 surgeries, *p*<0.001) compared to the overall rate from 2011 to 2017 (3.5 infections per 100 surgeries) (**Figure 4**), which was an outlier attributable to two hospitals where investigations are ongoing. Since 2018, the rate decreased by 48% from 7.5 to 3.9 infections per 100 surgeries in 2020, returning to rates observed prior to 2018.

**Table 4:** Paediatric cardiac surgical site infection rates by year and infection type, 2011–2020

Year	Superficial incisional SSI cases		Organ/space SSI cases		Deep incisional SSI cases		All cases^a^
	n	%	n	%	n	%	
2011	8	53.3	5	33.3	2	13.3	15
2012	15	83.3	2	11.1	1	5.6	18
2013^b^	12	66.7	6	33.3	0	0.0	18
2014	11	57.9	8	42.1	0	0.0	19
2015	12	66.7	5	27.8	1	5.6	18
2016	9	64.3	3	21.4	2	14.3	14
2017	17	70.8	5	20.8	2	8.3	24
2018	18	46.2	15	38.5	6	15.4	40
2019	16	51.6	13	41.9	2	6.5	31
2020	29	78.4	6	16.2	2	5.4	37
Overall	147	63.1	68	29.2	18	7.7	234

Abbreviation: SSI, surgical site infection

^a^ Excludes cases with missing infection type information

^b^ Excludes one site with missing denominator data (number of cases=0 in that year)

The median age of patients with a paediatric cardiac SSI was 19 days (IQR=7–193 days), and the median time from surgery to onset date of infection was 10 days (IQR=5–19 days). Among the four deaths reported within 30 days of infection onset (1.7% of cases), two deaths were unrelated to the paediatric cardiac SSI, while two were attributable to the paediatric cardiac SSI. *Staphylococcus aureus* and CoNS were the most commonly identified pathogens from paediatric cardiac SSIs (48% and 22% of identified pathogens, respectively) and did not differ by superficial, organ/space or deep infection type (data not shown).

### Antibiogram

Results of antimicrobial susceptibility testing for the most frequently identified gram-positive, gram-negative and fungal pathogens from device and surgical procedure-related HAIs are listed in **Table 5** and **Table 6**. The S. aureus isolates were resistant to cloxacillin/oxacillin (methicillin-resistant *S. aureus* [MRSA]) in 15% (n=32/218) of CLABSIs and 14% (n=40/284) of other reported SSIs. Meropenem resistance ranged from 2%–7% in gram-negative pathogens identified from CLABSIs. No meropenem resistance was observed among pathogens isolated from SSIs. Fifty-one vancomycin-resistant *Enterococci* were identified among CLABSIs (16%).

**Table 5:** Antibiogram results^a^ from pathogens identified from central line-associated bloodstream infections, 2015–2020

Antibiotic	Number of resistant/number tested and %															
	Gram-positive						Gram-negative						Fungi			
	Coagulase-negative staphylococci^b^		*S. aureus* ^c^		*Enterococcus* spp.		*Klebsiella* spp.		*E. coli*		*Enterobacter* spp.		*C. albicans*		*Candida* spp. other^d^	
	# of resistant	%	# of resistant	%	# of resistant	%	# of resistant	%	# of resistant	%	# of resistant	%	# of resistant	%	# of resistant	%
Ampicillin	13/15	87	N/A	N/A	126/368	34	119/122	98	71/112	63	60/64	94	N/A	N/A	N/A	N/A
Cefazolin	167/193	87	16/120	13	N/A	N/A	35/95	37	29/92	32	55/56	98	N/A	N/A	N/A	N/A
Ceftriaxone	15/19	79	4/12	33	N/A	N/A	16/100	16	13/84	15	37/65	57	N/A	N/A	N/A	N/A
Clindamycin	159/305	52	31/126	25	N/A	N/A	N/A	N/A	N/A	N/A	N/A	N/A	N/A	N/A	N/A	N/A
Ciprofloxacin	N/A	N/A	N/A	N/A	N/A	N/A	11/105	10	22/76	29	1/86	1	N/A	N/A	N/A	N/A
Cloxacillin/Oxacillin	306/351	87	32/218	15	N/A	N/A	N/A	N/A	N/A	N/A	N/A	N/A	N/A	N/A	N/A	N/A
Erythromycin	77/91	85	17/64	27	N/A	N/A	N/A	N/A	N/A	N/A	N/A	N/A	N/A	N/A	N/A	N/A
Gentamicin	20/39	51	1/25	4	13/109	12	9/128	7	13/109	12	7/92	8	N/A	N/A	N/A	N/A
Meropenem	17/18	94	N/A	N/A	N/A	N/A	4/59	7	1/42	2	1/64	2	N/A	N/A	N/A	N/A
Piperacillin-tazobactam	N/A	N/A	N/A	N/A	3/13	23	11/99	11	14/88	16	25/66	38	N/A	N/A	N/A	N/A
Penicillin	105/106	99	58/65	89	6/22	27	N/A	N/A	N/A	N/A	N/A	N/A	N/A	N/A	N/A	N/A
Rifampin	2/64	3	0/20	0	N/A	N/A	N/A	N/A	N/A	N/A	N/A	N/A	N/A	N/A	N/A	N/A
Trimethoprim-sulfamethoxazole	91/183	50	4/102	4	0/1	0	13/102	13	37/84	44	12/69	17	N/A	N/A	N/A	N/A
Tobramycin	N/A	N/A	N/A	N/A	N/A	N/A	7/106	7	4/99	4	4/77	5	N/A	N/A	N/A	N/A
Vancomycin	0/28	0	1/114	1	51/313	16	N/A	N/A	N/A	N/A	N/A	N/A	N/A	N/A	N/A	N/A
Amphotericin B	N/A	N/A	N/A	N/A	N/A	N/A	N/A	N/A	N/A	N/A	N/A	N/A	0/24	0	0/18	0
Caspofungin	N/A	N/A	N/A	N/A	N/A	N/A	N/A	N/A	N/A	N/A	N/A	N/A	0/35	0	1/56	2
Fluconazole	N/A	N/A	N/A	N/A	N/A	N/A	N/A	N/A	N/A	N/A	N/A	N/A	1/107	1	24/93	26

Abbreviations: *C. albicans*, *Candida albicans*; *E. coli*, *Escherichia coli*; N/A, not available; *S. aureus*, *Staphylococcus aureus*

^a^ Antibiotic/organism combinations with fewer than six tests were excluded

^b^ Coagulase-negative staphylococci included *S. lugdunensis, S. haemolyticus, S. epidermidis, S. capitis, S. hominis* and *S. warneri*

^c^ Included methicillin-susceptible *S. aureus* and methicillin-resistant *S. aureus*

^d^ Other *Candida spp.* included *C. dubliniensis*, *C. glabrata*, *C. krusei*, *C. lusitaniae*, *C. parapsilosis*, and *C. tropicalis*

**Table 6:** Antibiogram results^a^ from pathogens identified from paediatric cardiac, cerebrospinal shunt fluid and hip and knee surgical site infections^b^, 2015–2020

Antibiotic	Number of resistant/number tested and %															
	Gram-positive						Gram-negative						Fungi			
	Coagulase-negative staphylococci^c^		*S. aureus* ^d^		*Enterococcus* spp.		*Klebsiella* spp.		*E. coli*		*Enterobacter* spp.		*C. albicans*		*Candida spp.* other^e^	
	# of resistant	%	# of resistant	%	# of resistant	%	# of resistant	%	# of resistant	%	# of resistant	%	# of resistant	%	# of resistant	%
Ampicillin	N/A	N/A	N/A	N/A	1/42	2	6/6	100	11/19	58	16/20	80	N/A	N/A	N/A	N/A
Cefazolin	41/61	67	21/159	13	N/A	N/A	N/A	N/A	4/17	24	18/18	100	N/A	N/A	N/A	N/A
Ceftriaxone	N/A	N/A	N/A	N/A	N/A	N/A	N/A	N/A	3/10	30	8/17	47	N/A	N/A	N/A	N/A
Clindamycin	18/77	23	43/212	20	N/A	N/A	N/A	N/A	N/A	N/A	N/A	N/A	N/A	N/A	N/A	N/A
Ciprofloxacin	1/7	14	3/24	13	N/A	N/A	0/8	0	6/17	35	0/19	0	N/A	N/A	N/A	N/A
Cloxacillin/Oxacillin	80/133	60	40/284	14	N/A	N/A	N/A	N/A	N/A	N/A	N/A	N/A	N/A	N/A	N/A	N/A
Erythromycin	20/48	42	35/105	33	N/A	N/A	N/A	N/A	N/A	N/A	N/A	N/A	N/A	N/A	N/A	N/A
Gentamicin	N/A	N/A	0/15	0	5/14	36	2/9	22	4/20	20	1/23	4	N/A	N/A	N/A	N/A
Meropenem	N/A	N/A	N/A	N/A	N/A	N/A	N/A	N/A	0/9	0	0/7	0	N/A	N/A	N/A	N/A
Piperacillin-tazobactam	N/A	N/A	N/A	N/A	N/A	N/A	N/A	N/A	1/7	14	6/11	55	N/A	N/A	N/A	N/A
Penicillin	13/16	81	52/56	93	N/A	N/A	N/A	N/A	N/A	N/A	N/A	N/A	N/A	N/A	N/A	N/A
Rifampin	0/27	0	2/53	4	N/A	N/A	N/A	N/A	N/A	N/A	N/A	N/A	N/A	N/A	N/A	N/A
Trimethoprim-sulfamethoxazole	19/69	28	2/198	1	N/A	N/A	0/6	0	3/15	20	1/17	6	N/A	N/A	N/A	N/A
Tobramycin	N/A	N/A	N/A	N/A	N/A	N/A	1/8	13	1/16	6	0/19	0	N/A	N/A	N/A	N/A
Vancomycin	0/96	0	1/114	1	0/24	0	N/A	N/A	N/A	N/A	N/A	N/A	N/A	N/A	N/A	N/A
Amphotericin B	N/A	N/A	N/A	N/A	N/A	N/A	N/A	N/A	N/A	N/A	N/A	N/A	N/A	N/A	N/A	N/A
Caspofungin	N/A	N/A	N/A	N/A	N/A	N/A	N/A	N/A	N/A	N/A	N/A	N/A	N/A	N/A	N/A	N/A
Fluconazole	N/A	N/A	N/A	N/A	N/A	N/A	N/A	N/A	N/A	N/A	N/A	N/A	N/A	N/A	N/A	N/A

Abbreviations: *C. albicans*, *Candida albicans*; *E. coli*, *Escherichia coli*; N/A, not available; *S. aureus*, *Staphylococcus aureus*

^a^ Antibiotic/organism combinations with fewer than six tests were excluded

^b^ Antibiogram data collection for HK SSI began in 2016

^c^ Coagulase-negative staphylococci included *S. lugdunensis, S. haemolyticus, S. epidermidis, S. capitis, S. hominis* and *S. warneri*

^d^ Included methicillin-susceptible *S. aureus* and methicillin-resistant *S. aureus*

^e^ Other *Candida spp.* included *C. dubliniensis*, *C. glabrata*, *C. krusei*, *C. lusitaniae*, *C. parapsilosis*, and *C. tropicalis*

## Discussion

This report summarizes 4,751 device and surgical procedure-related HAIs identified over 10 years of surveillance from 2011 to 2020. Rates of device and surgical procedure-related HAIs have doubled for adult mixed ICU CLABSIs while NICU CLABSI and knee SSI rates have significantly decreased 60% and 58%, respectively. The most frequently reported pathogens in this report were generally aligned with those reported in a 2020 United States (US) National Healthcare Surveillance Network (NHSN) report of adult HAIs, indicating *S. aureus*, *E. coli* and *Klebsiella* among the most frequently reported pathogens for device and surgical procedure-related HAIs in both Canada and the US, while CoNS was identified more commonly in Canada ((9)). The COVID-19 pandemic may have had differing impacts on the rates of device and surgical procedure-related HAIs in Canada and the US ((10)). Investigation is underway to assess the influence of pandemic-related factors such as changes in infection control practices, hospital resource capacity, screening, laboratory testing and antimicrobial stewardship on the observed rates of HAIs.

### Central line-associated bloodstream infections

The overall rates of CLABSI in adult ICUs (0.6 and 1.1 per 1,000 line days for CVICUs and mixed ICUs, respectively) were similar to those reported in the US and Australia. The 2013 CLABSI rate in US medical/surgical ICUs was estimated to be 0.8 per 1,000 line days ((11)). In Australia, annual rates of CLABSIs in adult ICUs ranged between 0.9 and 1.4 CLABSIs per 1,000 line days from 2011–2013 ((12)). While CLABSI rates in adult mixed ICUs, CVICUs and PICUs have increased or remained stable in Canada since 2011, rates in NICUs have decreased by 60%. Data available from the US since 2016 indicate similar trends for CLABSIs in neonatal critical care locations, where the standardized incidence ratios (defined as the ratio of observed number of infections compared to the 2015 baseline) decreased by 27% ((13–17)). These decreased CLABSI rates in the US may be attributed to the updated NHSN guidelines for the prevention of CLABSI, implemented in 2011 ((18,19)).

Higher rates of CLABSIs are seen in other regions; a large surveillance study of intensive care units in 45 countries from Latin America, Europe, Eastern Mediterranean, Southeast Asia and Western Pacific World Health Organization regions reported pooled mean CLABSI rates of 7.2 per 1,000 line days in PICUs, 5.1 in medical/surgical adult ICUs and 12.0 in NICUs (between January 2012 and December 2017) ((11)).

### Surgical site infections

Among SSIs included in this surveillance report, hip and knee SSIs were the most common. Hip SSI rates remained stable across the reported years, while a decreasing trend in knee SSI rates was observed. Surveillance from the European Centre for Disease Prevention and Control reported similar trends, indicating stable hip SSI rates and decreasing knee SSI rates for study years 2014 to 2017 ((20)). In a US point prevalence study, a reduction in the prevalence of complex SSIs was observed between 2011 and 2015 ((21)). In accordance with pathogen results from other regions, the most common pathogens among hip and knee-SSIs were *S. aureus* and CoNS ((20,22)). Frequent identification of these two pathogens may be attributable to the use of implant devices and contamination from the patient’s endogenous skin flora ((9)). Joint replacements typically occur in older adults, which explains the high median age for hip and knee SSI ((23)). Joint replacements among older populations are also prone to surgical complications, such as prosthetic joint infections ((23)). Data indicate that surgical site infections frequently lead to readmission and revision surgery, both of which result in high financial and resource burdens on the healthcare system ((24)).

The overall rate of surgical site infections from CSF shunts was 2.9 per 100 surgeries. This aligns with rates reported from a 2012 multi-country review, which range from 3% to 12% ((25)). Stratification of CSF shunt SSI data by paediatric and adult/mixed hospitals showed that adult rates (2.8/100 surgeries) and paediatric rates (3.0/100 surgeries) were similar from 2011–2020. Data from a previous CNISP study conducted between 2000 and 2002 indicated a higher paediatric rate than the adult rate of CSF shunt SSI ((26)). Given that the rate of CSF shunt SSI among paediatric patients from 2011–2020 (3.0%) is lower than that from 2000–2002 (4.9%), there is evidence of a decrease in SSI rates among paediatric populations ((26)). Meanwhile, the rate of CSF shunt SSI among adult patients from 2011–2020 (2.8%) remains relatively unchanged compared to that of 2000–2002 (3.2%) ((26)).

The overall rate of paediatric cardiac SSI between 2011 and 2020 was 4.1 per 100 surgeries. The 2018 paediatric cardiac SSI rate should be interpreted with caution; given that the number of cases used to calculate this rate was limited, the rates may be sensitive to fluctuation attributed to individual hospital sites. Nevertheless, the overall rate was found to be comparable with infection rates reported elsewhere, despite limited literature about paediatric cardiac SSIs. A 2009–2012 intervention study of neonates undergoing cardiac surgery at a New York tertiary-care centre found pre and post-intervention paediatric cardiac SSI rates of 6.2 and 5.8/100 surgeries, respectively ((27)). In France, 19% of patients younger than one year of age and undergoing cardiac surgery presented with a SSI during the study period, between 2012 and 2013 ((28)). The hospital-acquired cardiac-SSI rate at two New York hospitals was 1.4 infections per 100 procedures within 90 days for patients younger than 18 years of age, based on a retrospective study from 2010–2012 ((29)).

### Antibiogram

The percentage of *S. aureus* isolates that were MRSA in this study (14%–15%) (Table 5 and Table 6) was slightly higher to what was reported from a Swiss surveillance network where 8% of *S. aureus* SSI cases were MRSA in 2010–2015 ((30)). Higher rates of MRSA have been reported elsewhere, such as in several centres in Latin America where resistance averaged 44.7% in 2017 ((31)). In the US, 42%–48% of *S. aureus* isolates from HAIs (including SSI, CLABSI and others) in NHSN surveillance were MRSA ((9)).

Of the identified *Enterococcus* spp. in CLABSIs, 16% were vancomycin-resistant *Enterococci,* which is less than 30.9% identified as resistant in ICUs in Poland ((32)). From NHSN surveillance in the US, 84.5% of *Enterococcus faecium* and 8.5% of *Enterococcus faecalis* pathogens identified from CLABSIs in ICUs were vancomycin-resistant *Enterococci* in 2015–2017 ((9)).

Meropenem resistance was low among the gram-negative pathogens identified among CLABSIs and SSIs (0%–7%). Similarly in the US, the percent of carbapenem resistance among *Klebsiella* spp. ranged from 3.1% (among SSIs) to 6.9% (among expanded list of device-associated infections); the percent of carbapenem resistance among *E. coli* ranged from 0.6% (among SSIs) to 0.7% (expanded list) ((9))*.*

### Strengths and limitations

The main strength of this study is the standardized collection of detailed data from a large network of sentinel hospitals for over ten years. The CNISP network extends across Canada, although it may not be representative of all Canadian acute care hospitals since the number of hospitals participating in each HAI surveillance project differed. However, recruitment is ongoing and CNISP coverage of Canadian acute care beds increased from 25% in 2011 to 30% in 2020. The CNISP is continuing to increase representativeness, especially among northern, community, rural and Indigenous populations.

The epidemiologic data collected were limited to the information available in the patient charts. For CLABSI surveillance, data were limited to infections occurring in the ICU settings, and as such may only represent a portion of CLABSIs occurring in the hospital. Further, differences in surveillance protocols and case definitions, as well as the lack of recent comparable data, limit comparison with data from other countries. The CNISP continues to support the national public health response to the COVID-19 pandemic. Future studies are ongoing to assess the impact of the COVID-19 pandemic on device and surgical procedure-related HAIs and AMR.

## Conclusion

This report provides an updated summary of rates, pathogen distributions and antimicrobial resistance among select device and surgical procedure-related HAIs and relevant pathogens. The collection and analysis of national surveillance data are key to understanding and reducing the national burden of device and surgical procedure-related HAIs by providing benchmark rates for comparison nationally and internationally and informing antimicrobial stewardship and infection prevention and control programs and policies.

## Appendix Case definitions

### Central line-associated bloodstream infection

Only central line-associated bloodstream infections (BSIs) related to an intensive care unit (ICU) admission were included in surveillance.

Bloodstream infections case definition:

Bloodstream infection is **NOT** related to an infection at another site and it meets one of the following criteria:

**Criterion 1:** Recognized pathogen cultured from at least one blood culture, unrelated to infection at another site.

OR

**Criterion 2:** At least one of: fever (higher than 38°C core), chills, hypotension; if aged younger than 1 year, fever (higher than 38°C core), hypothermia (lower than 36°C core), apnea or bradycardia **AND** common skin contaminant (see list below) cultured from at least two blood cultures drawn on separate occasions or at different sites, unrelated to infection at another site. Different sites may include peripheral veins, central venous catheters or separate lumens of a multilumen catheter. Different times include two blood cultures collected on the same or consecutive calendar days via separate venipunctures or catheter entries. The collection date of the first positive blood culture is the date used to identify the date of positive culture. Two positive blood culture bottles filled at the same venipuncture or catheter entry constitute only one positive blood culture.

Central line-associated bloodstream infection case definition:

A central line-associated bloodstream infections (CLABSI) must meet one of the following criteria:

**Criterion 1:** A laboratory-confirmed bloodstream infection (LCBSI) where a central line catheter (CL) or umbilical catheter (UC) was in place for more than two calendar days on the date of the positive blood culture, with day of device placement being Day 1.

OR

**Criterion 2:** A LCBSI where a CL or UC was in place more than two calendar days and then removed on the day or one day before positive blood culture was drawn.

Intensive care unit-related central line-associated bloodstream infection case definition:

A CLABSI related to an ICU if it meets one of the following criteria:

**Criterion 1:** CLABSI onset after two days of ICU stay.

OR

**Criterion 2:** If the patient is discharged or transferred out of the ICU, the CLABSI would be attributable to the ICU if it occurred on the day of transfer or the next calendar day after transfer out of the ICU.

Note: If the patient is transferred into the ICU with the CL and the blood culture was positive on the day of transfer or the next calendar day, then the CLABSI would be attributed to the unit where the line was inserted.

Common skin contaminants:

Diphtheroids, *Corynebacterium* spp., *Bacillus* spp., *Propionibacterium* spp., coagulase-negative staphylococci (including *S. epidermidis*), viridans group streptococci, *Aerococcus* spp., *Micrococcus* spp. and *Rhodococcus* spp.

### Hip and knee surgical site infection

Only complex surgical site infections (SSIs) (deep incisional or organ/space) following hip and knee arthroplasty were included in surveillance.

A deep incisional surgical site infection must meet the following criterion:

Infection occurs within 90 days after the operative procedure and the infection appears to be related to the operative procedure and involves deep soft tissues (e.g. facial and muscle layers) of the incision and the patient has at least **ONE** of the following:

- Purulent drainage from the deep incision but not from the organ/space component of the surgical site
- Deep incision that spontaneously dehisces or is deliberately opened by the surgeon and is culture-positive or not cultured when the patient has at least one of the following signs or symptoms: fever (higher than 38°C) or localized pain or tenderness (a culture-negative finding does not meet this criterion)
- An abscess or other evidence of infection involving the deep incision is found on direct examination, during reoperation or by histopathologic or radiologic examination
- Diagnosis of a deep incisional SSI by a surgeon or attending physician

An organ/space surgical site infection must meet the following criterion:

Infection occurs within 90 days after the operative procedure and the infection appears to be related to the operative procedure and infection involves any part of the body, excluding the skin incision, fascia or muscle layers, that is opened or manipulated during the operative procedure and patient has at least **ONE** of the following:

- Purulent drainage from a drain that is placed through a stab wound into the organ/space
- Organisms isolated from an aseptically obtained culture of fluid or tissue in the organ/space
- An abscess or other evidence of infection involving the organ/space that is found on direct examination, during reoperation or by histopathologic or radiologic examination
- Diagnosis of an organ/space SSI by a surgeon or attending physician

### Cerebrospinal fluid shunt surgical site infection

Only patients who underwent a placement or revision of a cerebrospinal fluid (CSF) shunting device and the infection occurred within one year of surgery were included in surveillance.

Cerebrospinal fluid shunt-associated surgical site infection case definition:

An internalized CSF shunting device is in place **AND** a bacterial or fungal pathogen(s) is identified from the cerebrospinal fluid **AND** is associated with at least **ONE** of the following:

- Fever (temperature 38°C or higher)
- Neurological signs or symptoms
- Abdominal signs or symptoms
- Signs or symptoms of shunt malfunction or obstruction

### Paediatric cardiac surgery surgical site infection

Only surgical site infections following open-heart surgery with cardiopulmonary bypass among paediatric patients (younger than 18 years of age) were included in surveillance.

A **superficial incisional SSI** must meet the following criterion: Infection occurs within 30 days after the operative procedure and involves only skin and subcutaneous tissue of the incision and meets at least **ONE** of the following criteria:

- Purulent drainage from the superficial incision
- Organisms isolated from an aseptically obtained culture of fluid or tissue from the superficial incision
- At least **ONE** of the following signs or symptoms of infection:
  - Pain or tenderness, localized swelling, redness or heat, and the superficial incision is deliberately opened by a surgeon, and is culture-positive or not cultured (a culture-negative finding does not meet this criterion)
  - Diagnosis of superficial incisional SSI by the surgeon or attending physician

A **deep incisional SSI** must meet the following criterion:

Infection occurs within 90 days after the operative procedure and the infection appears to be related to the operative procedure **AND** involves deep soft tissues (e.g. facial and muscle layers) of the incision **AND** the patient has at least **ONE** of the following:

- Purulent drainage from the deep incision but not from the organ/space component of the surgical site
- Deep incision spontaneously dehisces or is deliberately opened by the surgeon and is culture-positive or not cultured when the patient has at least one of the following signs or symptoms: fever (higher than 38**°**C) or localized pain or tenderness (a culture-negative finding does not meet this criterion)
- An abscess or other evidence of infection involving the deep incision is found on direct examination, during reoperation or by histopathologic or radiologic examination
- Diagnosis of a deep incisional SSI by a surgeon or attending physician

An **organ/space SSI** must meet the following criterion:

Infection occurs within 90 days after the operative procedure and the infection appears to be related to the operative procedure **AND** infection involves any part of the body, excluding the skin incision, fascia or muscle layers, that is opened or manipulated during the operative procedure **AND** the patient has at least **ONE** of the following:

- Purulent drainage from a drain that is placed through a stab wound into the organ/space
- Organisms isolated from an aseptically obtained culture of fluid or tissue in the organ/space
- An abscess or other evidence of infection involving the organ/space that is found on direct examination, during reoperation or by histopathologic or radiologic examination

**Table A1:** Rate of central line-associated bloodstream infection per 1,000 line days by intensive care unit type, 2011–2020

Year	Adult Mixed ICU	Adult CVICU	NICU	PICU
2011	0.8	0.8	4.0	1.2
2012	0.9	0.9	3.3	1.1
2013	0.9	0.6	3.2	1.0
2014	0.8	0.5	2.2	1.7
2015	1.0	0.7	2.3	2.1
2016	1.0	0.3	2.3	1.6
2017	1.1	0.3	1.8	1.6
2018	1.1	0.8	1.8	1.9
2019	1.4	0.6	2.0	1.8
2020	1.6	0.7	1.6	1.7
Overall	1.1	0.6	2.3	1.6

Abbreviations: CLABSI, central line-associated bloodstream infection; CVICU, cardiovascular intensive care unit; ICU, intensive care unit; NICU, neonatal intensive care unit; PICU, paediatric intensive care unit

**Table A2:** Rate of hip and knee surgical site infections per 100 surgeries, 2011–2020

Year	Hip	Knee
2011	0.82	0.69
2012	0.73	0.65
2013	0.79	0.41
2014	0.85	0.56
2015	0.74	0.43
2016	0.79	0.35
2017	0.78	0.34
2018	0.88	0.31
2019	0.70	0.30
2020	0.48	0.29
Overall	0.79	0.45

**Table A3:** Cerebrospinal fluid shunt surgical site infection rates per 100 surgeries by hospital type, 2011–2020

Year	Adult and Mixed hospitals	Paediatric hospitals	All hospitals^a^
2011	4.60	5.66	5.20
2012	2.21	3.08	2.70
2013	2.47	2.40	2.43
2014	0.84	2.12	1.36
2015	3.44	1.91	2.54
2016	4.19	2.00	2.93
2017	4.17	2.74	3.41
2018	1.93	1.46	1.70
2019	3.13	5.13	3.96
2020	2.42	3.21	2.80
Overall	2.84	2.96	2.90

^a^ All hospitals include adult, mixed, and paediatric hospitals participating in cerebrospinal fluid shunt surgical site infection surveillance

**Table A4:** Paediatric cardiac surgical site infection rates per 100 surgeries, 2011–2020

Year	Rate
2011	3.13
2012	2.90
2013	4.32
2014	3.45
2015	3.27
2016	3.02
2017	4.43
2018	7.46
2019	5.47
2020	3.90
Overall	4.14
